# Papillome inverse de la vessie: une tumeur bénigne rare: à propos d'un Cas

**DOI:** 10.11604/pamj.2017.26.204.12266

**Published:** 2017-04-13

**Authors:** Hicham El Bote, Sihem Atik, Rami Fares, Ernest Hage

**Affiliations:** 1 Service d'Urologie A, CHU Ibn Sina, Rabat, Maroc; 2 Service d’Oncologie médicale, hôpital central de l’armée HCA, Alger, Algérie; 3 Service d’Urologie, Centre hospitalier de Soissons, Soissons, France

**Keywords:** Papillome inversé, tumeur, vessie, traitement, suivi, Inverted papilloma, tumor, bladder, treatment, follow-up

## Abstract

Le papillome inversé de la vessie est une tumeur urothéliale rare et bénigne, qui apparaît généralement à l’âge adulte avec une nette prédominance masculine. L’hématurie macroscopique est le symptôme révélateur le plus fréquent, les signes radiologiques et cystoscopiques ne sont pas toujours spécifiques. Son diagnostic de certitude repose sur l’examen anatomopathologique. Le traitement est basé sur la résection endoscopique avec coagulation du lit tumoral. Une surveillance au long cours est nécessaire en raison de son association relativement fréquente avec le carcinome urothélial.

Inverted papilloma of the bladder is a rare and benign urothelial tumor, which usually affects adult patients with a clear male predominance. Macroscopic haematuria is the most frequent revealing symptom. Radiologic and cystoscopic signs are not always specific. Definitive diagnosis is based on anatomo-pathological examination. Treatment is based on endoscopic resection with coagulation of the tumor bed. Long term monitoring is necessary due to its common association with urothelial carcinoma.

## Introduction

Le papillome inversé de la vessie est une tumeur bénigne rare représentant 1 à 2.2% des tumeurs vésicales, son diagnostic est anatomopathologique. Le traitement est basé sur la résection endoscopique; son suivi est nécessaire du fait de sa fréquente association aux carcinomes urothélials. La rareté de cette localisation nous incite à rapporter cette observation.

## Patient et observation

**Figure 1:**
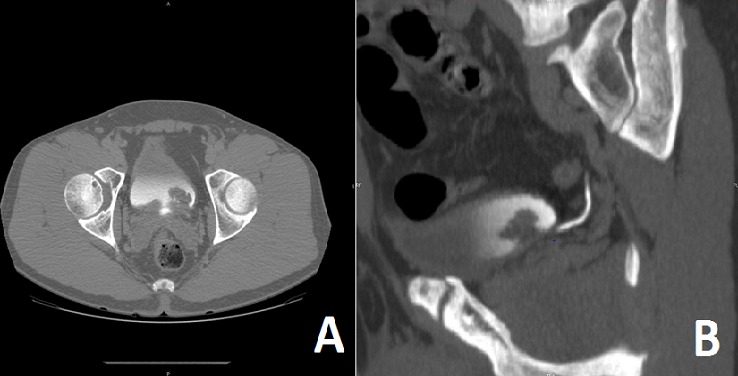
Uroscanner (temps excréteur): processus tissulaire (2.6 x 2.6 mm) au niveau de la partie postéro-latérale gauche de la vessie: A) coupe axiale; B) coupe sagittale

**Figure 2:**
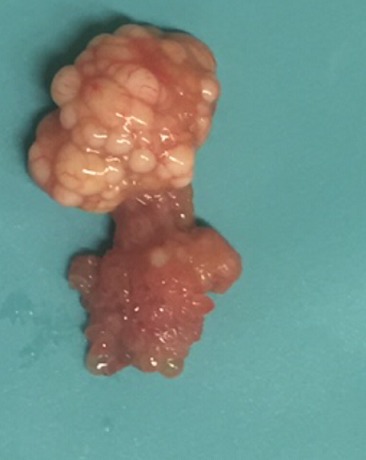
Aspect macroscopique: lésion polypoïde atypique non papillaire, à surface lisse d’environ 4 cm

**Figure 3:**
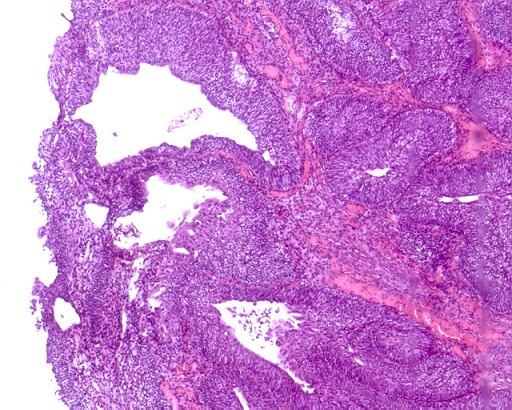
Examen anatomopathologique: revêtement épithélial bien différencié a tendance à former des papilles ou des structures kystiques

Nous rapportons le cas d'un patient âgé de 49 ans, tabagique chronique 20 Paquet-Année, ayant comme antécédent pathologique un asthme depuis l’enfance sous traitement bien équilibré et une appendicectomie à l’âge de 7 ans, présentant un épisode d’hématurie macroscopique totale associée à des signes irritatifs du bas appareil urinaire sans retentissement hémodynamique, le tout évoluait dans un contexte d'apyrexie et de conservation de l'état général. L'examen clinique trouvait un patient en bon état général, un abdomen souple, des fosses lombaires libres et un toucher rectal normal. L'uroscanner avait révélé une un processus tissulaire (2.6 x 2.6 mm) au niveau de la partie postéro-latérale gauche de la vessie sans dilatation des voies excrétrices en amont (Figure 1). La cytologie urinaire était négative. La résection transurétrale a permis l'exérèse en monobloc d’une tumeur vésicale polypoïde atypique non papillaire, à surface lisse d’environ 4 cm rétro-méatique gauche (Figure 2). L'examen anatomopathologique (Figure 3) et immuno-histochimique (P53 et Ki67) ont conclu à un papillome inversé. Le malade a bénéficié d’une cystoscopie à 3 mois revenue normale dans le cadre de sa surveillance.

## Discussion

Initialement décrit par Paschkis en 1927 [1] et nommé Par Potts et Hirst en 1963 [2], le papillome inversé de la vessie est une tumeur bénigne et rare qui représente entre 1 et 2.2% des tumeurs urothéliales [3, 4]. Depuis 1963, environ 600 cas ont été reporté. Aucune étiologie n’est retenue jusqu’à l’heure actuelle, Cummings [5] et Matz et al. [6] ont suggéré que cette lésion n'était pas néoplasique mais plutôt une sorte de réaction hyperplasique, secondaire à une inflammation chronique ou à des agents irritants. Le papillome inversé de la vessie apparaît généralement dans la cinquième et la sixième décennie de la vie avec un âge moyen de 59 ans, la prédominance masculine est importante avec un sex ratio allant de 5 à 8 [4]. L’hématurie macroscopique est le symptôme révélateur le plus fréquent puisqu’elle est présente dans plus de 50% des cas. Elle peut être isolée ou associée à des signes d’irritation vésicale. La cytologie urinaire est habituellement négative [7]. L'échographie montre une masse vésicale solide, échogène à surface régulière, parfois polypoïde, dont elle précise la taille et le caractère pédiculé ou non. L’aspect cystoscopique typique est sous la forme d’une tumeur non papillaire, non invasif, à surface lisse, polypoïde pédiculé ou sessile, qui peut se développer sur tout l’urothélium de la vessie, particulièrement au niveau du trigone et du col vésical, qui restent la localisation préférentielle avec une fréquence de 84.5% [8]. Le diagnostic de certitude repose sur l’examen anatomopathologique. En 1975, Henderson et al. ont établi les critères diagnostiques histologiques du papillome inversé, y compris la prolifération cellulaire endophytique, la présence de cellules transitionnelles normales au niveau de l’épithélium de revêtement et de cellules uniformes sans atypies cytonucléaires au niveau de la lésion avec des mitoses absentes ou rares, ainsi que la présence de microkystes à contenu éosinophile et rarement de petits foyers de métaplasie épidermoide [9]. En 1983, Kunze et al. ont distingué deux types histologiques du papillome inversé : trabéculaire et glandulaire [10]. Le principal diagnostic différentiel est le carcinome urothélial, incluant une néoplasie de faible potentiel de malignité ou un carcinome urothélial de bas grade. D'autres diagnostics différentiels sont plus rares, comprenant l'adénome néphrogénique, le paragangliome, la tumeur carcinoïde et les Cystites [11]. Le traitement est basé sur la résection endoscopique avec coagulation du lit tumoral. Billery et al. [12] ont affirmé que le papillome inversé est la seule tumeur urothéliale dont on est assurée du caractère constamment bénin et non récidivant. L’association d’un carcinome urothélial au papillome inversé a été décrite et n’est pas rare [13], imposant une surveillance au long cours. En 2013, Picozzi et al ont recommandé de réaliser une cystoscopie souple tous les 4 mois dans la première année et ensuite tous les 6 mois pendant les 3 années suivantes. La surveillance systématique du haut appareil urinaire n'est pas jugée nécessaire [4].

## Conclusion

Le papillome inversé de la vessie est une tumeur bénigne et rare, qui reste relativement méconnue par les urologues. Il se manifeste par des signes cliniques, radiologiques et endoscopiques généralement non spécifiques. Son diagnostic de certitude repose sur l’examen anatomopathologique et son association avec les carcinomes urothéliaux impose une surveillance rigoureuse après sa résection endoscopique.
